# Enhanced strand transfer and mismatch extension by HIV-1C reverse transcriptase promote sequence motif duplication

**DOI:** 10.1128/jvi.00526-26

**Published:** 2026-06-04

**Authors:** Arun Panchapakesan, Aditya Pradeepbhai Joshi, Afzal Amanullah, Neelam Pargain, Kavita Mehta, Manisenthil Shanmugam, Jayendra Singh, Chhavi Saini, Narendra Nala, Udupi A. Ramagopal, Udaykumar Ranga

**Affiliations:** 1 HIV-AIDS Laboratory, Molecular Biology and Genetics Unit, Jawaharlal Nehru Centre for Advanced Scientific Research 29130, Bengaluru, Karnataka, India; 2 Molecular Biology Laboratory, Y. R. Gaitonde Centre for AIDS Research and Education (YRGCARE), Chennai, Tamil Nadu, India; 3 Flow Cytometry Facility, Molecular Biology and Genetics Unit, Jawaharlal Nehru Centre for Advanced Scientific Research 29130, Bengaluru, Karnataka, India; 4 Division of Biological Sciences, Poornaprajna Institute of Scientific Research, Bidalur, Bengaluru, India; 5 Department of Microbiology and FST, School of Science, GITAM University 28668, Visakhapatnam, India; The Ohio State University, Columbus, Ohio, USA

**Keywords:** HIV-1C, reverse transcriptase, nonhomologous recombination, sequence duplication

## Abstract

Genetic diversification of HIV-1 is driven largely by the error-prone activity of reverse transcriptase (RT) and the high frequency of template switching during reverse transcription. A rare consequence of nonhomologous recombination is sequence motif duplication, which can alter viral gene regulation and protein function. Previous studies have shown that such duplications occur at higher frequencies in HIV-1C, particularly within the long terminal repeat and p6-Gag regions, where they can confer replication advantages. However, the mechanistic basis for this subtype-specific bias has remained unclear. Here, we investigated whether intrinsic biochemical properties of HIV-1C RT contribute to its elevated duplication frequency. Bioinformatic analysis of 6,877 full-length HIV-1 genomes identified four duplication hotspots across the viral genome, with the highest frequencies in HIV-1C. Comparative sequence analysis revealed several subtype-specific residues in RT, including a conserved threonine at position 359 (T359) in the connection domain. Structural modeling suggested that T359 can form an additional hydrogen bond with the nascent cDNA, potentially stabilizing the RT–template complex. Biochemical characterization of recombinant RT variants demonstrated that residue 359 modulates polymerase activity and maintains subtype-specific optimal catalytic function. Functional assays further showed that HIV-1C RT exhibits enhanced template strand transfer compared with HIV-1B RT. In addition, next-generation sequencing–based primer extension assays revealed that HIV-1C RT extends mismatched 3′ termini more efficiently across multiple mismatch types. Together, these findings indicate that subtype-specific biochemical properties of HIV-1C RT, mediated in part by T359, promote nonhomologous recombination events that generate sequence motif duplications.

**IMPORTANCE:**

High-frequency sequence motif duplications are a distinctive feature of HIV-1C, yet the molecular mechanisms underlying their higher prevalence have remained unclear. Here, we demonstrate that intrinsic biochemical properties of HIV-1C reverse transcriptase (RT) promote these events through enhanced template strand transfer and improved extension of mismatched 3′ termini during reverse transcription. These properties increase the probability of nonhomologous recombination events that generate these duplications across the viral genome. Importantly, such duplications have major biological consequences at hotspots. In the long terminal repeat, duplication of transcription factor binding motifs produces promoter variants that alter transcriptional strength, influencing both latency establishment and reversal. In the p6-Gag region, duplication of the PTAP motif is linked to compensatory adaptation in drug resistance and may enhance viral fitness under antiretroviral pressure. Together, our findings provide a mechanistic explanation for the emergence of clinically relevant viral variants and highlight how subtle subtype-specific RT polymorphisms can shape HIV-1 evolution and persistence.

## INTRODUCTION

The extraordinary genetic diversity of human immunodeficiency virus type 1 (HIV-1), which enables its rapid adaptation and persistence in the host, is largely driven by the viral reverse transcriptase (RT). Unlike most cellular polymerases, HIV-1 RT lacks 3′–5′ exonuclease proofreading, leading to frequent nucleotide misincorporation during DNA synthesis, although its intrinsic error rate is similar to that of other retroviral RTs (1). However, the exceptionally high rate of recombination of the RT elevates HIV-1′s genetic diversity to levels among the highest observed in retroviruses (2).

Recombination, an essential feature of retroviral replication, typically occurs between homologous regions of co-packaged RNA genomes during reverse transcription. A small proportion (0.1%–1%) of recombination events, however, arises through nonhomologous pathways involving microhomology domains (3). In such cases, the site at which RT dissociates from the donor RNA and the site at which it resumes synthesis on the acceptor RNA are not identical, resulting in sequence duplications, deletions, or insertion–deletion events (2).

Among these outcomes, sequence duplications are particularly intriguing because they can modulate viral gene expression, protein structure, and replication capacity. Although most duplication events are likely deleterious and eliminated by purifying selection, those that confer a fitness advantage can persist and expand within viral populations. Recurrent duplications have been reported in specific regions of the HIV-1 genome, most notably within the long terminal repeat (LTR) and the p6-Gag region, and are associated with enhanced replicative fitness (4–6). For instance, duplication of the NF-κB motif in the LTR enhances transcriptional activity (4), whereas duplication of the PTAP motif in p6-Gag can compensate for deleterious immune-escape or drug resistance mutations (5–7). Strong selective pressures on these regions have therefore rendered them prominent duplication “hotspots” in HIV-1 sequence databases.

Sequence duplications may also influence antiretroviral drug resistance. Several studies have documented higher frequencies of PTAP duplications among drug-resistant HIV-1 strains (8–11). In HIV-1 subtype C (HIV-1C), PTAP duplication frequencies are higher in both drug-naïve (23%) and treatment-failure (54%) cohorts compared to subtypes B and F (9). The PTAP duplication has been proposed to enhance proteolytic processing at the NC-Sp2-p6 junction, thereby compensating for fitness deficits imposed by resistance mutations (5).

Notably, motif duplications in both the LTR and gag regions occur more frequently in HIV-1C than in other subtypes. Among full-length viral genomes in extant databases, LTR duplications are observed in 27.3% of HIV-1B sequences and 34.5% of HIV-1C sequences (4, 12). Similarly, PTAP duplications are present in 16.9% of HIV-1B and 30.4% of HIV-1C sequences (6, 7). These findings raise the possibility that HIV-1C has an intrinsic propensity to generate sequence motif duplications at an enhanced rate, potentially reflecting subtype-specific differences in viral replication. The molecular basis for this disparity, however, remains unclear.

Because recombination is a prerequisite for sequence duplication, RT function is a logical focus for mechanistic investigation. HIV-1 RT mediates template strand transfer during reverse transcription, and its recombination profile is influenced by intrinsic properties such as polymerase and RNase H activities, processivity, and fidelity. For example, nucleotide misincorporation can slow polymerization and promote recombination (13–15). Although biochemical analyses suggest broadly similar enzymatic parameters for HIV-1B and HIV-1C RTs (16), studies using chimeric viral backbones have revealed subtype-specific differences in replication kinetics (17). Thus, even in the context of conserved biochemical properties, HIV-1C RT may employ distinct molecular mechanisms that favor higher frequencies of recombination and duplication. Sequence duplications, though relatively rare, offer a selective snapshot of these processes, as they are retained when they confer a replication advantage.

The present study addresses this gap by examining how subtype-specific polymorphisms in HIV-1C RT, particularly the T359 residue, may contribute to elevated sequence motif duplication. Through integrated bioinformatic, biochemical, and molecular analyses, we aim to define the mechanistic basis of sequence duplication in HIV-1C and to clarify its implications for viral evolution, replicative fitness, and drug resistance.

## RESULTS

### Four hotspots of sequence motif duplication exist in the HIV-1C genome

Our previous work identified hotspots of sequence motif duplication in two regions of the HIV-1C genome, the LTR and p6 (7, 12), prompting us to examine whether similar duplication hotspots occur elsewhere in the HIV-1C genome. We therefore retrieved 6,877 full-length HIV-1 genomes representing subtypes A1, B, C, D, F1, and G from the Los Alamos National Laboratory (LANL) HIV Sequence Database. Using a custom Python script, we mapped sequence duplications across these genomes. In total, we identified 389 distinct loci with sequence duplications, distributed across the genome, with an average inter-duplication distance of ~24.9 bp. Two notable exceptions were p24 and pol, where only 0 and two events were observed, respectively

**Fig 1:**
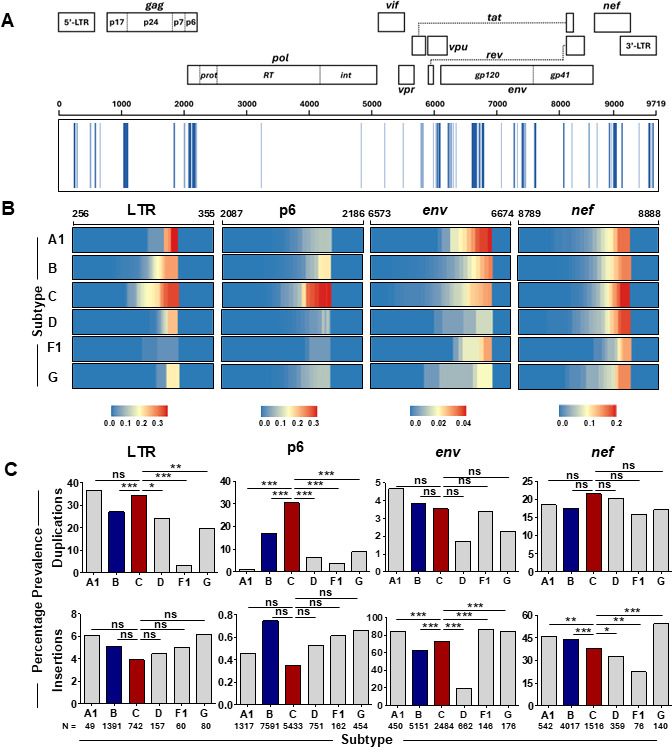
HIV-1C duplicates sequences at a higher frequency at hotspots. (**A**) Sequence duplications are observed throughout the HIV-1 genome. Full-length HIV-1 genome sequences (*n* = 6,877) representing all major subtypes were downloaded from the HIV-LANL database and analyzed using a custom Python script to identify duplication events. A duplication at a given location is shown using vertical blue lines. Genomic positions are indicated using HXB2 coordinates. (**B**) HIV-1C exhibits a high frequency of duplications across known hotspots. Sequences of the LTR, p6, env, and nef regions from subtypes A1, B, C, D, F1, and G were downloaded from the HIV-LANL database and screened for sequence duplications. The heatmap illustrates the per-base frequency of duplications across a 100 base pair region in each location, as depicted using HXB2 coordinates. (**C**) HIV-1C shows significantly higher duplication frequencies in the LTR and p6. Bar graphs showing the proportion of sequences in the database that contain duplications (top) and insertions (bottom) in the four hotspots in different subtypes. Subtypes B and C are highlighted. Statistical analysis was performed using Pearson’s Chi-square test. *P < 0.05, **P < 0.01, ***P < 0.001, *P < 0.05, **P < 0.01, and ***P < 0.001. *N* represents the number of sequences used in the analysis.

The apparent scarcity of duplications in p24 and pol is likely influenced by sampling bias in database-derived data sets. Because duplications frequently disrupt reading frames or impair protein function, such variants would be subject to strong negative selection *in vivo* and are therefore unlikely to be amplified or sequenced using standard approaches. Conversely, duplications that confer a selective advantage, such as those in the LTR and p6 regions, are more likely to persist and thus appear overrepresented. Consistent with this expectation, we observed a marked enrichment of duplications in these regions. Despite this limitation, the analysis indicates that duplications can arise throughout the HIV-1 genome but are preferentially concentrated at specific loci, likely reflecting selective advantages. In addition to the previously reported hotspots in the LTR and p6 regions, we identified two additional hotspots in the envelope and nef regions, where duplications were consistently observed across all subtypes (Fig. 1B and C).

Among the subtypes analyzed, HIV-1C showed the highest duplication frequencies in the LTR, p6, and nef regions, accounting for 34.5%, 30.5%, and 21.5% of duplications, respectively. The envelope region was an exception: subtypes A1 and B showed slightly higher duplication frequencies (4.6% and 3.8%) than HIV-1C (3.5%) (Fig. 1B and C). Overall, the average per-base duplication frequency across the four hotspots was markedly higher in HIV-1C than in other subtypes (Fig. 1B). However, only the LTR and p6 hotspots in HIV-1C showed statistical significance (Fig. 1C). One possible explanation is that the insertions in these two regions represent motif duplications that confer a replication advantage, as previously demonstrated (4–7, 12). Additionally, in both regions, sequence duplications represented the predominant class of insertions across all subtypes. On average, duplications accounted for 24.3% and 11.2% of sequences in the LTR and p6 regions, respectively, whereas other insertions were comparatively rare (5.1% and 0.5%) (Fig. 1C, bottom panel). In contrast, the envelope and nef regions showed the opposite pattern: although insertions were present, most did not represent duplication of adjacent sequence motifs (Fig. 1C, bottom panel). This suggests that insertions in these regions arise largely from stochastic variation rather than motif-specific duplication. Collectively, these analyses indicate that sequence motif duplication occurs more frequently in HIV-1C

### The T359 signature amino acid residue can form an additional hydrogen bond with the cDNA

To determine whether the higher frequency of sequence duplications in HIV-1C reflects subtype-specific features of RT, we performed a comparative analysis of HIV-1 RT sequences downloaded from the LANL database. We then performed Viral Epidemiology Signature Pattern Analysis (VESPA) (18) on 100 randomly selected RT sequences representing each of the major HIV-1 subtypes A1, B, C, and D. Subtypes F1 and G were excluded because of insufficient numbers of p66 sequences in the database. This analysis identified 28 amino acid residues specific to HIV-1C (Table 1). Applying a frequency threshold of >75% in HIV-1C and <25% in other subtypes reduced this set to six candidate residues: A36, T48, A200, Q245, T359, and R530. These residues span the fingers (A36 and T48), palm (A200), thumb (Q245), connection (T359), and RNase H (R530) domains of RT (Table 1).

**TABLE 1:** Frequencies of the signature amino acid residues in HIV-1C reverse transcriptase^*a*^

Domain	Position	HIV-1C			Other subtypes		
		Amino acid	Frequency	Non-HIV-1C amino acid frequency	Amino acid	Frequency	HIV-1C frequency
Fingers	36^*b*^	A	0.76	0.01	E	0.97	0.23
	39	E	0.62	0.02	T	0.58	0.01
	48^*b*^	T	0.93	0.02	S	0.98	0.05
	60	I	0.52	0.50	V	0.50	0.48
	123	G	0.42	0.04	D	0.43	0.29
Palm	173	A	0.68	0.04	K	0.48	0.03
	200^*b*^	A	0.96	0.21	T	0.53	0.01
	207	E	0.68	0.24	A	0.36	0.07
	211	K	0.62	0.34	S	0.40	0
Thumb	245^*b*^	Q	0.82	0.11	E	0.35	0.01
	272	P	0.8	0.45	A	0.46	0.15
	277	R	0.58	0.28	K	0.70	0.4
	291	D	0.94	0.43	E	0.56	0.06
	292	I	0.93	0.38	V	0.61	0.07
Connection	334	H	0.32	0.02	Q	0.84	0.29
	356	K	0.98	0.31	R	0.69	0.01
	357	M	0.76	0.28	K	0.43	0.01
	359^*b*^	T	0.87	0.03	S	0.48	0.05
	371	A	0.96	0.48	V	0.51	0.04
	377	M	0.46	0.16	T	0.52	0.03
	403	T	0.95	0.46	M	0.46	0
	404	D	0.96	0.31	E	0.69	0.04
RNase H	435	A	0.63	0.14	V	0.55	0.2
	452	I	0.51	0.06	L	0.88	0.22
	466	I	0.63	0.03	V	0.95	0.37
	480	Q	0.97	0.49	H	0.49	0.01
	483	Q	0.75	0.03	H	0.44	0.04
	530^*b*^	R	0.92	0.08	K	0.92	0.08

^*a*^ HIV-1C RT sequences were subjected to signature amino acid residue analysis using the VESPA tool available at the HIV-LANL database with subtypes A1, B, and D as background. Subtypes F1 and G were not included in the analysis due to the low number of sequences available. The single-letter amino acid code is used.

^*b*^ Amino acid residues selected for subsequent analyses.

**Fig 2:**
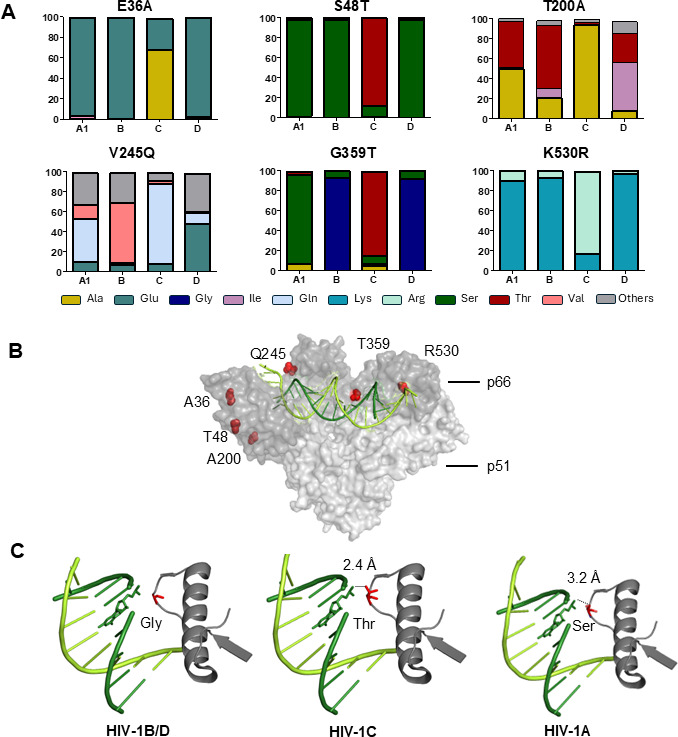
The T359 residue of HIV-1C forms a hydrogen bond with the cDNA. (**A**) Amino acid variation at six signature residues in HIV-1C reverse transcriptase. Amino acid frequencies at six key positions identified through sequence analysis. The reference amino acids indicated in the panel headings correspond to HXB2 and Indie-C1, respectively. (**B**) Spatial proximity of the T359 residue to the DNA strand in HIV-1 RT. A high-resolution structure of HIV-1B RT (PDB ID: 5J2M) was visualized using PyMOL. The six residues of interest are depicted in red as enlarged spheres. The DNA template and cDNA strands are shown in light and dark green, respectively. The p66 and p51 subunits are colored dark and light gray, respectively, and are shown in the background. (**C**) Hydrogen bond formation between the T359 residue and cDNA. The structural environment around residue 359 was examined using the same RT structure. The native glycine (left), and modified threonine (center) and serine substitutions (right) at this position were modeled. The cDNA base involved in hydrogen bonding is shown as sticks. The distance between the hydroxyl group of the residue and the phosphate group of the cDNA was measured in PyMOL and is represented by a dotted line.

Among these positions, T359 was of particular interest for three reasons. First, its presence in HIV-1C is exclusive to this subtype, as is that of two other residues at positions 36 and 48. In contrast, the three corresponding residues at the other three positions: 200, 245, and 530, occurred at comparatively lower frequencies in other subtypes (Fig. 2A and Table S1). Second, the glycine-to-threonine substitution at position 359 is non-conservative, introducing increased polarity and a potential for hydrogen bond formation. Four residues, alanine, glycine, serine, and threonine, occur at this position across subtypes with distinct distributions (Fig. 2A and Table S1). Glycine predominates in subtypes B and D (92.3% and 91.2%), serine in A1 (88.8%), whereas threonine is the dominant residue in HIV-1C (83.7%) and is nearly absent from other subtypes. Third, structural mapping of the six residues onto the RT structure showed that T359 lies near the nucleic acid-binding cleft, suggesting potential interaction with the DNA/RNA substrate (Fig. 2B).

To evaluate this possibility, we modeled serine and threonine substitutions using the HIV-1B RT crystal structure (PDB 5J2M) as a template. To validate our model, we further utilized AlphaFold 3 to generate a high-resolution model of the HIV-1C RT structure in complex with an RNA-DNA hybrid and ATP as a ligand (19). A structural overlay of the Connection domains (residues 319–426) from the experimental template and the AlphaFold 3-predicted model yielded a C-α root mean square deviation (RMSD) of 0.263 Å. This exceptionally low deviation confirms that the backbone architecture is highly conserved across these subtypes and that our template-based model is in complete agreement with predictive modeling.

Our model revealed that in subtypes B and D, which contain glycine at position 359, the absence of a side chain prevents strong interactions with DNA. In contrast, in subtypes A1 and C, which harbor serine and threonine, respectively, the hydroxyl groups of these residues lie in close proximity to a phosphate atom on the nascent cDNA backbone, enabling potential hydrogen-bond formation. The predicted atomic distances are ~3.2 Å for serine and ~2.4 Å for threonine (Fig. 2C). The shorter distance for threonine suggests a stronger interaction and supports a possible role for this residue in modulating RT-template contacts in HIV-1C.

### The T359 residue ensures an optimal RT activity in HIV-1C

Sequence and structural analyses indicated that the threonine residue at position 359 of HIV-1C RT is highly conserved and can form an additional hydrogen bond with the nascent DNA, suggesting a potential effect on polymerase function. Because polymerase activity influences downstream processes such as strand transfer, recombination, and sequence duplication, we examined how variation at residue 359 affects diverse RT functions.

To address this question, we constructed two panels of RT variants using HIV-1B and HIV-1C RTs as backbones. Each panel contained three enzymes differing only at residue 359, which carried the naturally occurring amino acids glycine, serine, or threonine (Fig. 2A). Recombinant RTs were generated by expressing the p66 and p51 subunits separately in *E. coli* M15 cells. The proteins were purified by sequential Ni-NTA and ion-exchange chromatography and assembled *in vitro* for biochemical analyses.

**Fig 3:**
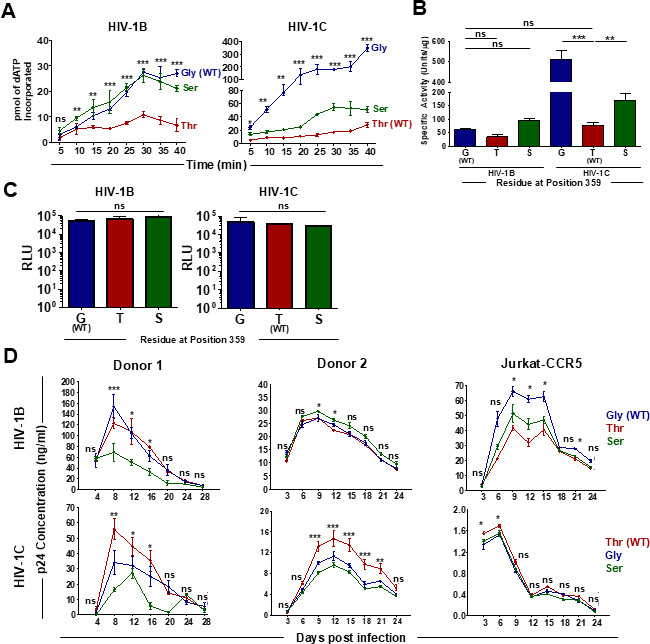
Polymerase function of the RT variants. (**A**) cDNA synthesis rate of RT variants. cDNA synthesis rates of HIV-1B (left) and HIV-1C (right) RT variants expressed and purified from *E. coli*. A homopolymeric template, polyuridylic acid, was extended with a primer, and ³²P-dATP incorporation was monitored by liquid scintillation at 5-min intervals. Data are presented as Mean ± SD and are representative of three independent experiments. Note the difference in the y-axis between subtypes. Data were analyzed by two-way ANOVA with Bonferroni post hoc test. (**B**) Specific activities of the RT variant proteins. The amount of radioactive dATP incorporated in 10 min was used to calculate the specific activity of all RT variants. The data are representative of three independent experiments, and are plotted as mean ± SD, followed by one-way analysis of variance and Tukey-Kramer post hoc tests. (**C**) Single-round infectivity of RT variant viral strains. Replication-competent NL4-3 (HIV-1B) and Indie (HIV-1C) clones carrying Gly, Ser, or Thr at RT position 359 were used for the assay. Viral stock titers were measured by infecting TZM-bl cells with 10 ng p24 of each variant. Luciferase induction by Tat-driven LTR activity served as a surrogate for viral replication. Data represent mean ± SD (*n* = 3), analyzed by one-way ANOVA with Tukey–Kramer post hoc test. (**D**) Replication profiles of RT variant viral strains. Replication kinetics of RT-variant viral strains in Jurkat-CCR5 T-cells and CD8-depleted PBMCs from two donors. Cells (3 × 10^6^) were infected with 10 ng p24 equivalent of each of the viral stocks, and viral proliferation was estimated by measuring the p24 production at 3- or 4-day intervals using a commercial kit. The amino acid color code is consistent across the figure. The data, represented as mean ± SD, were analyzed by two-way ANOVA with Bonferroni post hoc test. ***P < 0.001, **P < 0.01, and *P < 0.05, ns = not significant.

Polymerase activity was measured by ^32^P-dATP incorporation on a poly-U template over a kinetic time course. All six enzymes were active and showed progressive incorporation of the isotope (Fig. 3A). However, clear subtype-dependent differences in polymerase kinetics emerged. HIV-1B RTs (B-RTs) containing glycine or serine, the most common residues at this position in this subtype, displayed substantially higher activity than the threonine-containing variant. At 30 min, glycine- and serine-containing B-RTs incorporated 27.47 ± 1.11 and 26.26 ± 2.85 pmol dATP, respectively, whereas the threonine variant incorporated only 10.81 ± 1.16 pmol. In contrast, HIV-1C RT (C-RT) containing the canonical T359 showed the lowest catalytic activity, which increased markedly upon substitution. After 30 min, activity rose from 17.1 ± 1.79 pmol in the T359 enzyme to 179.28 ± 7.66 pmol and 54.18 ± 5.19 pmol with glycine and serine substitutions, respectively (Fig. 3A). Thus, RTs carrying the canonical residue at position 359 for their subtype exhibited comparable activity; substitution with a non-canonical residue reduced activity in HIV-1B (threonine) but markedly increased it in HIV-1C (glycine).

Subtype-specific differences were also evident in specific activity across the RT panel, consistent with previous reports (16, 17). HIV-1B and HIV-1C RTs containing their canonical residues (G359 and T359, respectively) showed comparable activities of 64.98 ± 5.33 and 76.15 ± 12.24 U/µg (Fig. 3B). In B-RT, substitution with serine moderately increased activity (95.24 ± 8.23 U/µg), whereas threonine reduced it to 34.65 ± 9.64 U/µg. In contrast, substitutions in C-RT produced striking increases: the canonical T359 enzyme (76.15 ± 12.24 U/µg) rose to 510.66 ± 43.39 U/µg (6.7-fold) and 170.25 ± 27.23 U/µg (2.23-fold) with glycine or serine substitutions, respectively (Fig. 3B). These findings indicate that variation at position 359 strongly influences C-RT function but has only modest effects on B-RT.

The increase in catalytic activity observed upon substituting threonine with glycine or serine in C-RT suggests that T359 normally imposes a regulatory constraint on HIV-1C RT. The formation of an additional hydrogen bond between T359 and the nascent cDNA may slow catalytic kinetics, thereby maintaining activity within an optimal range. Consistent with this model, replacing threonine with serine, which forms a weaker hydrogen bond due to its longer bond length (~3.2 Å), resulted in only a modest increase in activity. In HIV-1, reverse transcription typically requires 8–33 hours depending on the host cell type (20); excessively rapid DNA synthesis could therefore compromise replication fitness. Notably, RT sequences vary substantially among HIV-1 subtypes. For example, the RTs of NL4-3 (HIV-1B) and Indie (HIV-1C), used in our assays, differ by 7.68% at the amino-acid level. Such intrinsic variation may confer higher catalytic potential to C-RT, which T359 may help restrain to prevent excessively rapid reverse transcription.

Because the T359G variant of HIV-1C RT showed the highest catalytic activity among the enzymes tested, we next evaluated the effects of residue 359 substitutions in replication-competent viruses. Viral panels analogous to the recombinant RT panels (Fig. 3A) were generated using the NL4-3 (HIV-1B) and Indie-C1 (HIV-1C) molecular clones. Viral stocks produced in HEK293T cells showed comparable infectious titers across variants, as measured by TZM-bl luciferase assays following infection with 10 ng p24 (Fig. 3C). Replication kinetics were then assessed in primary PBMCs from two healthy donors and in Jurkat-CCR5 cells. All variants replicated efficiently, with peak p24 production between days 6 and 12 irrespective of the backbone (Fig. 3D). As expected, NL4-3-based viruses generally produced higher p24 levels than Indie-C1 derivatives.

Within each panel, the virus carrying the subtype-specific canonical residue at position 359 replicated most efficiently (Fig. 3D). In the NL4-3 panel, the G359 strain (wild-type HIV-1B) reached 153.90 ± 39.63 ng/mL p24 at day 8 in donor 1 PBMCs, whereas the T359 and S359 variants produced 122.88 ± 18.15 and 69.09 ± 28.03 ng/mL, respectively. Similarly, in the Indie-C1 panel, the wild-type T359 virus produced 55.95 ± 12.09 ng/mL p24 at day 8, compared with 34.13 ± 13.64 and 16.25 ± 2.07 ng/mL for the G359 and S359 variants. Replication patterns were broadly consistent in donor 2 PBMCs and Jurkat-CCR5 cells, although modest donor-specific differences were observed, particularly in the NL4-3 panel. Thus, although variant HIV-1C RTs with non-canonical amino acid residues at position 359 showed higher catalytic activity than the enzyme with the canonical threonine residue, in the context of the infectious viral clone, the viral strain with the canonical threonine at position 359 demonstrated the highest level of proliferation, consistent with our model.

Together, these results demonstrate that residue 359 plays a key role in determining RT catalytic function. Canonical residues at this position, glycine in HIV-1B and threonine in HIV-1C, maintain optimal RT activity and are not interchangeable between subtypes. Notably, these residues occur at the highest frequencies within their respective subtypes and are rarely shared between them. Combined with the biochemical data, these findings suggest that efficient viral replication requires RT activity to remain within a defined functional window. Our results further indicate that T359 represents a subtype-specific regulatory adaptation in HIV-1C, moderating RT kinetics to maintain optimal activity and preserve replication fitness.

### HIV-1C RT mediates enhanced strand transfer *in vitro*

Because recombination is a prerequisite for sequence duplication, a higher recombination frequency could explain the greater prevalence of motif duplications in HIV-1C. Given the central role of residue T359 in HIV-1C RT and the likelihood that this residue forms an additional hydrogen bond with the nascent cDNA, we hypothesized that threonine may stabilize the RT-cDNA complex and thereby enhance recombination efficiency. However, previous experimental work using an HIV-1C molecular clone (21) reported findings inconsistent with this hypothesis. Importantly, that study used a subgenomic viral backbone, highlighting the need to reassess the model using a near-full-length viral construct.

**Fig 4:**
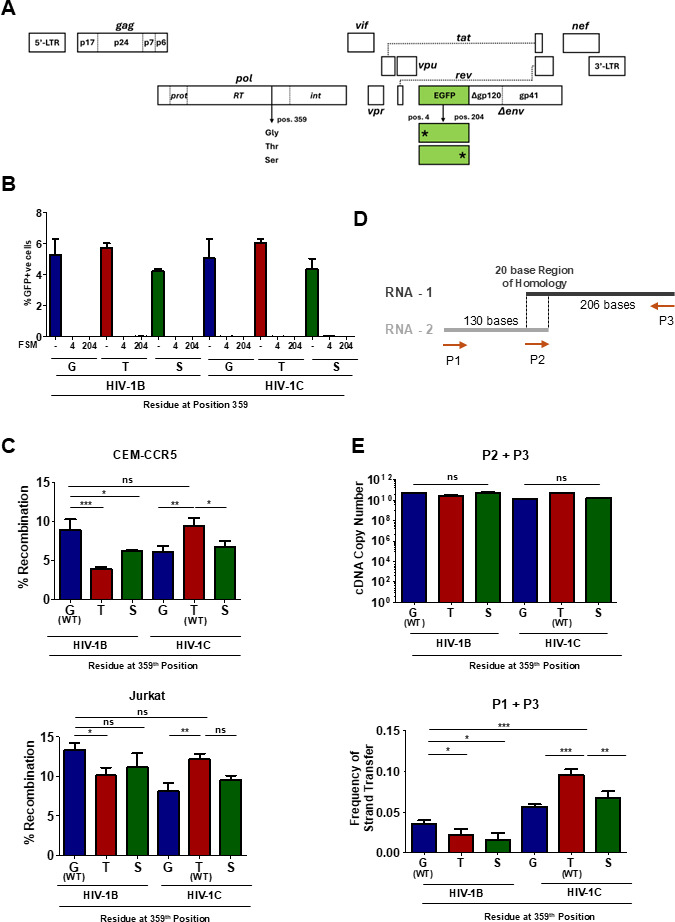
HIV-1B and -1C RTs recombine at comparable frequencies. (**A**) Schematic of the EGFP reporter virus design. Panels of three RT variants (glycine, threonine, or serine at position 359) were generated using NL4-3 or Indie near full-length molecular clones. The viral envelope was replaced with an EGFP ORF, allowing single-round infection when pseudotyped. The EGFP reading frame contains frame-shift mutations (FSM) at amino acid positions 4 or 204, resulting in a total of 18 viral strains. (**B**) Homozygous GFP mutant viruses do not express GFP. Viral stocks of all 18 strains were produced in HEK293T cells and used to infect CEM-CCR5 or Jurkat cells. After 24 h, cells were activated with a cocktail of TNF-α (10 ng/mL), HMBA (10 ng/mL), and PMA (5 mM). GFP expression was assessed 24 h post-activation. The X-axis shows the position of the frameshift mutation. WT = wild-type EGFP. (**C**) Recombination frequencies of wild-type RTs. Viral stocks with FSM4 and FSM204 EGFP mutations were used in different combinations to infect CEM-CCR5 or Jurkat cells, as shown in Table S2. GFP expression was quantified as the percentage of GFP+ cells among infected cells. (**D**) Strand-transfer assay schematic. Two *in vitro*-transcribed RNA templates with a 20 bp overlap (dark/light gray lines) and primers (orange arrows) were used. Ct values were obtained via SYBR Green-based qPCR. (**E**) HIV-1C RT variants show enhanced strand transfer. cDNA copy numbers (primers P2 + P3) were determined by regression (left) and used to normalize values of strand transfer PCR (P1 + P3, right). Data are shown as mean ± SD from three independent experiments. Statistical significance was assessed using one-way ANOVA with Tukey–Kramer post hoc test. ***P < 0.001, **P < 0.01, and *P < 0.05, ns = not significant.

To evaluate the effect of residue 359 on recombination, we employed a fluorescent EGFP reporter recombination assay (22, 23). This assay measures template switching by quantifying cells expressing functional EGFP restored from two complementary frame-shifted defective EGFP precursors. Two viral panels were generated in the NL4-3 (HIV-1B) and Indie-C1 (HIV-1C) backbones, each containing glycine, serine, or threonine at position 359 (Fig. 4A). In total, 18 pseudotypable, replication-competent viral constructs were produced (Fig. 4A and Table S2). Because the assay uses a single-round infection system, only one cycle of infection occurs, preventing additional rounds of recombination. We first confirmed that only constructs carrying an intact EGFP cassette, but not those containing premature termination codons, produced detectable fluorescence after transfection into HEK293 cells (Fig. 4B).

Following co-infection with viruses encoding the two defective EGFP templates, the proportion of GFP-positive cells reflected the recombination capacity of each RT variant. Wild-type HIV-1B and HIV-1C RTs exhibited similar recombination frequencies in CEM-CCR5 cells, 8.86% ± 1.36% and 9.42% ± 0.94%, respectively (Fig. 4C, top panel), consistent with earlier reports (21). Substitution of the canonical residue in either subtype significantly reduced recombination efficiency. In HIV-1B, replacing glycine at position 359 with threonine or serine reduced GFP-positive cells to 3.91% ± 0.21% and 6.14% ± 0.15%, respectively. Similarly, in HIV-1C, replacing the canonical threonine with glycine or serine yielded 6.09% ± 0.73% and 6.72% ± 0.73% GFP-positive cells.

A similar pattern was observed in Jurkat T cells, although overall recombination frequencies were slightly higher. For example, the GFP-positive fraction for the G359 HIV-1B RT decreased from 13.38% ± 0.85% to 10.1% ± 1.0% and 11.16% ± 1.77% after substitution with threonine or serine (Fig. 4C, bottom panel). Likewise, replacing the canonical threonine in HIV-1C RT reduced GFP-positive cells from 12.02% ± 0.69% to 8.13% ± 0.99% and 9.56% ± 0.53%, respectively. These findings are consistent with the dynamic copy-choice model, in which faster reverse transcription correlates with reduced template switching.

Since sequence duplication requires RT to switch between the two genomic RNA templates, we next asked whether HIV-1C RT intrinsically promotes template switching more efficiently. To test this, we performed a template-switching assay using two single-stranded RNA templates with a 20-base overlap (Fig. 4D, top panel). Successful strand transfer during reverse transcription produces a longer 336 bp cDNA product detectable by PCR, whereas the absence of strand transfer yields a shorter 206 bp fragment. The assay was performed using both HIV-1B and HIV-1C RT panels (Fig. 4E). All six RT variants generated comparable levels of the 206 bp product that does not require template switching (Fig. 4E, middle panel). However, marked differences, both between and within subtypes, were observed in the formation of the 336 bp product requiring strand transfer (Fig. 4E, bottom panel).

Notably, all HIV-1C RT variants collectively exhibited higher strand-transfer activity than HIV-1B RTs. Within HIV-1C, the canonical T359 enzyme showed the highest switching frequency (0.096 ± 0.01), compared with the glycine and serine variants: 0.057 ± 0.00 and 0.07 ± 0.01, respectively (Fig. 4E, bottom panel). In contrast, HIV-1B RT variants displayed substantially lower activity, with glycine (wild type), threonine, and serine variants showing switching frequencies of 0.03 ± 0.00, 0.03 ± 0.00, and 0.02 ± 0.01, respectively. These results indicate that HIV-1C RT possesses intrinsically higher strand-transfer capacity, a key mechanistic driver of sequence duplication. Collectively, the data support a model in which T359 enhances template engagement through an additional hydrogen bond, whereas substitutions that weaken this interaction impair recombination-related functions. Thus, threonine at position 359 appears to represent a subtype-specific regulatory adaptation that fine-tunes reverse transcription dynamics to support the elevated duplication frequencies characteristic of HIV-1C.

### HIV-1C RT extends 3′-OH mismatches more efficiently

**Fig 5:**
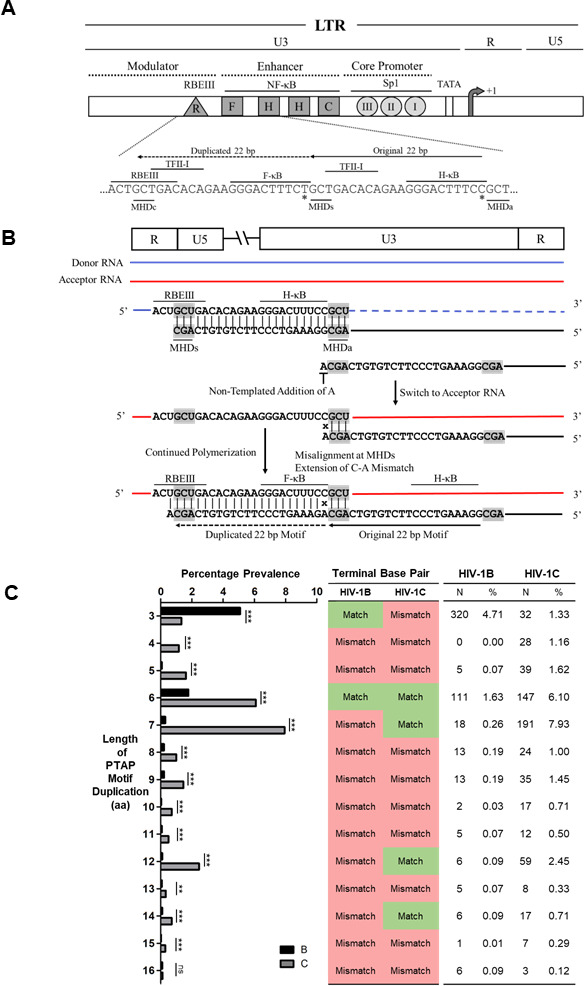
Schematic representation of the 22 bp NF-κB motif duplication in HIV-1C mediated by extension of a mismatched base. (**A**) Organization of the HIV-1C LTR showing the relative positions of the four NF-κB motifs and other key transcription factor–binding sites (TFBSs), along with their nucleotide sequences. Solid arrows indicate the original sequence, whereas dashed arrows denote the duplicated segment. The 3-base pair repeats flanking the original sequence, which is recreated in the duplication, called the Micro-homology domain (MHD), are underlined. The three such MHD triplets corresponding to positions where the RT stalls, re-aligns, and creates a new copy are designated MHDs, MHDa, and MHDc, respectively. Asterisks mark the C-to-T variation distinguishing the H-κB and F-κB motifs. The duplicated 22 bp segment corresponds to positions 325–352 of the HXB2 reference sequence. Image reproduced from reference 24, previously published under a Creative Commons CC BY 4.0 license. (**B**) Schematic showing the template-switching mechanism and mismatch extension. Donor and acceptor RNA templates are depicted in blue and red, respectively, while the nascent cDNA is shown in black. Dashed lines represent RNA regions degraded by the RNase H activity of RT. The extended mismatched base is indicated by an “x” MHD motifs are shaded in gray. (**C**) Spikes in sequence motif duplication are associated with perfect base pairing of the terminal base pair. Comparative analysis of PTAP duplication lengths in HIV-1B and HIV-1C. Gag sequences from the LANL database were classified by duplication length, and the prevalence of each length is shown as the percentage of sequences for HIV-1B (*N* = 6,797, shown in black) and HIV-1C (*N* = 2,401, shown in gray). The middle panel indicates the presence or absence of a perfect base-pair match between nascent cDNA and the acceptor RNA (color-coded), and the right panel summarizes the number and percentage of sequences with each duplication. Statistical analysis was performed using Pearson’s chi-square test.

The phenomenon of sequence motif duplication requires that RT switch to the acceptor RNA template at a location that has already been copied from the donor RNA. However, such an erroneous strand switch, although very rare, imposes a constraint when it occurs, because the sequences at the growing end of the nascent cDNA may be discordant, thereby hampering efficient resumption of polymerization. Notably, retroviral RTs can anneal to mismatched sites using very short regions of complementarity, sometimes as few as three bases or fewer, termed micro-homology domains (MHDs), to continue reverse transcription. Such MHD-mediated annealing is predicted to be highly transient and unstable, making nonhomologous recombination events exceedingly rare (3). Furthermore, following MHD-mediated hybridization, a base-pair mismatch at the 3′ end of the nascent cDNA is expected to occur in three out of four instances, requiring RT to efficiently extend a mispaired terminus to resume polymerization and complete the duplication event. For example, in HIV-1C, the LTR typically contains three NF-κB-binding sites, designated H, H, and C, where “H” denotes sites conserved across HIV-1 subtypes, and “C” denotes the subtype C–specific motif. Notably, one of the H NF-κB sites is frequently duplicated to generate a fourth NF-κB motif, termed the F site. Importantly, the “F-κB” site differs from the canonical “H-κB” site at the 10th position, where there is a “C to T” change (Fig. 5A). Examination of the sequence context underlying this duplication reveals the presence of a conserved MHD on either side of the duplicated motif. To generate the F-κB site, the RT pauses at the 5′ ‘G’ of the GCT motif, which serves as the stall site, designated MHDs, after reverse transcription of the upstream H-κB, TFII-I, and partial RBEIII elements (Fig. 5B). The paused RT then adds a non-templated nucleotide, preferentially an “A”, to the growing cDNA strand. Subsequent strand transfer allows the nascent DNA to anneal either correctly to a homologous acceptor sequence, resulting in standard recombination, or, less frequently, to a misaligned GCT motif (the acceptor MHD, called MHDa) on the acceptor RNA. This misalignment generates a C:A mismatch at the 3′ end of the nascent DNA, which is nevertheless extended by RT. Such a mismatch extension stabilizes the duplicated segment and introduces the characteristic “C to T” transition at position 10 of the newly formed F-κB site ([24], Fig. 5B).

However, there is no direct evidence on how mismatched bases influence sequence duplication frequencies. Analysis of PTAP motif duplications in p6-Gag provided an opportunity to examine this question, given the substantial variation in duplication length observed in natural sequences. We previously reported that PTAP motif duplication is highly polymorphic, displaying variation in duplication length, sequence divergence between the original and duplicated segments, and subtype-associated patterns (6). For example, HIV-1B preferentially duplicates only three residues (APP) of the six-amino-acid PTAP core motif (EPTAPP), although the biological significance of this partial duplication remains unclear. In contrast, HIV-1C more frequently duplicates six or seven amino acids (EPTAPPA), thereby reproducing the core motif in full. Nevertheless, both subtypes can generate duplications up to 16 amino acids in length (Fig. 1). In HIV-1C, additional but smaller peaks are also observed at 9, 12, and 14 amino acid duplications (6, 7). Although these data demonstrate subtype-specific preferences in duplication length, the presence of multiple peaks within each subtype suggests that strand-transfer efficiency alone cannot fully account for the observed diversity.

To explore whether sequence features at the strand-switch junction influence duplication outcomes, we compared nucleotide sequences between the original and duplicated motifs. Our analysis identified locations on the donor RNA where the RT must stall, and the position on the acceptor RNA where it must misalign to generate the observed duplications. Importantly, since the duplication is non-homologous, we focused on the terminal base that the RT must extend on the cDNA after misalignment. This analysis revealed that peaks in duplication length strongly correlate with perfect base pairing between the 3′ end of the nascent cDNA and the terminal base of the acceptor RNA template (Fig. 5C). Furthermore, when three nucleotides of the cDNA form correct base pairs with the acceptor template, an efficient MHD is established, and the duplication frequency increases substantially. This pattern is consistently observed at all major duplication peaks in both HIV-1C (six, seven, twelve, and fourteen amino acids) and HIV-1B (three and six amino acids). Thus, complementarity at the 3′ terminus appears to facilitate efficient extension and completion of the duplication event.

In contrast, events requiring mismatch-dependent extension, particularly those lacking an MHD (four, five, eight, nine, ten, eleven, and thirteen amino-acid duplications), occurred at substantially higher frequencies in HIV-1C than in HIV-1B. For example, a nine-amino-acid PTAP motif duplication occurred at a frequency of 1.45% (35/2,401) in HIV-1C, compared with only 0.19% (13/6,797) in HIV-1B (Fig. 5C). These observations suggest that HIV-1C RT is more efficient at extending mismatched termini, thereby promoting motif duplication.

**Fig 6:**
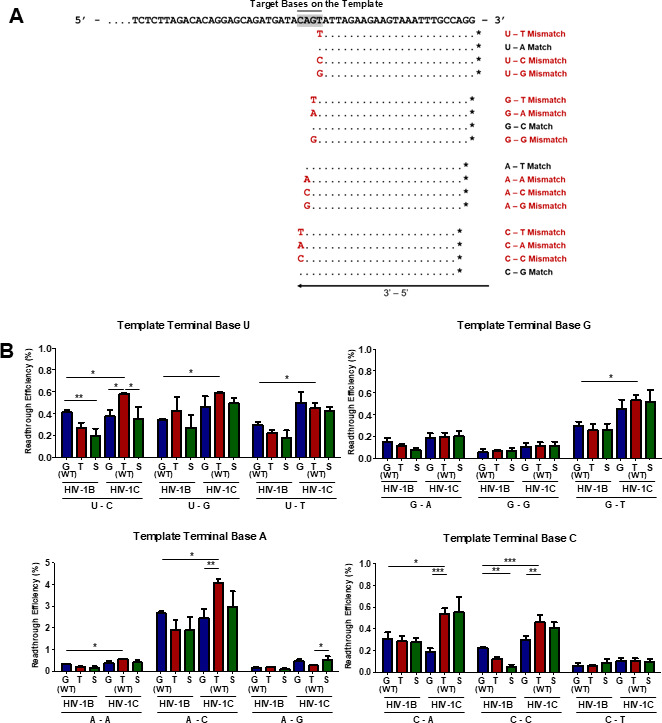
HIV-1C RT can extend mismatched bases more efficiently. (**A**) Schematic representation of the mismatch extension assay. A 331-nt region on the HIV-1 genome (HXB2: 1583–1914) was used as a template. Four primer sets containing four primers per set each were designed such that three primers in each set carried a distinct 3′ mismatch to represent the 12 possible mismatches; dots indicate complementary bases. The four base pairs where reverse transcription is initiated on the template by the four primer sets are highlighted in gray. An asterisk (*) indicates the unique 8 bp barcode for each primer. (**B**) HIV-1C RT is more efficient at extending mismatched bases. An RNA template was generated by *in vitro* transcription and annealed to 16 individual barcoded primers representing all base-pair combinations. Reverse transcription was performed with 1 U of the corresponding RT variant, and the efficiency of reverse transcription was estimated by Illumina sequencing. Readthrough efficiency was calculated by normalizing the read counts for the mismatched base pair combinations for each RT with the corresponding matched base read counts and is represented as a percentage. The terminal template base is shown above each panel, with the corresponding primer base indicated below the X-axis. RT variants are color-coded by the amino acid at position 359. Data represent mean ± SD from three independent experiments. Statistical analysis was performed by one-way ANOVA with Tukey–Kramer post hoc test. Statistical significance is shown only for WT comparisons and their corresponding variants and where test results are significant. ***P < 0.001, **P < 0.01, and *P < 0.05.

To test this hypothesis experimentally, we performed primer extension assays using recombinant RT panels representing HIV-1B and HIV-1C variants to determine whether HIV-1C RT extends mismatched termini more efficiently during reverse transcription. An RNA template corresponding to the PTAP motif region (331 nucleotides; HXB2 coordinates: 1,583–1,914) was transcribed *in vitro*. Within this template, we identified a stretch of four bases, “AUGC” (HXB2 coordinates: 1,868–1,872), where the growing end of primers could align with any one of these four bases (Fig. 6A). Four DNA primers were designed with identical sequences except for the terminal nucleotide, such that each primer ended with a different base (A, C, G, or T) at the 3′ end. In each primer set, one primer matched the target template base perfectly, whereas the remaining three represented mismatches. Four such primer sets were generated, each terminating opposite one base of the “AUGC” sequence. Each primer also contained a unique barcode for NGS analysis. In total, the reverse transcription assay employed sixteen primers and three RT enzymes from each subtype panel (bearing G, S, or T at position 359), representing HIV-1B and HIV-1C variants.

Extension reactions were performed for all primer-template combinations using the RT variants from each panel. The extended products were subjected to second-strand synthesis and prepared for Illumina sequencing to quantify mismatch extension efficiency. Sequencing reads corresponding to each primer-template pair were enumerated, and readthrough efficiency was calculated as the proportion of products in which the mismatched 3′ terminus was successfully extended, normalized to the number of reads obtained for the corresponding matched control primer for each RT variant.

Mismatch extension was observed under all experimental conditions, with readthrough efficiencies ranging from 0.06% to 4.09% (Fig. 6B). Consistent with previous reports, purine-purine and pyrimidine-pyrimidine mismatches showed the lowest readthrough efficiencies across RT variants (25–28). In contrast, purine-pyrimidine mismatches displayed substantially higher readthrough efficiencies, with an average readthrough of 0.96% across variants. Among these, the A-C mismatch exhibited the highest readthrough potential, followed by U-G, C-A, and G-T.

Overall, HIV-1C RT variants exceeded HIV-1B RTs in extending mismatched bases under most experimental conditions, with many differences reaching statistical significance. The canonical T359 HIV-1C RT showed enhanced extension of eight of the twelve possible mismatch types (A-A, A-C, C-A, C-C, G-T, U-C, U-G, and U-T) compared with the canonical G359 HIV-1B RT. For example, when the terminal base on the template was “A” and the primer ended with a mismatched “C,” the readthrough efficiencies of the three HIV-1B RTs (G359, T359, and S359) were 2.69% ± 0.09%, 1.92% ± 0.45%, and 1.91% ± 0.59%, respectively. The corresponding efficiencies for HIV-1C RTs were 2.46% ± 0.41%, 4.09% ± 0.18%, and 2.97% ± 0.71%, respectively. Interestingly, HIV-1C RT variants exhibited higher readthrough efficiencies across all mismatch combinations when the template base was U. For U-C, U-G, and U-T mismatches, the readthrough efficiencies for wild-type B-RT were 0.41% ± 0.01%, 0.34% ± 0.01%, and 0.29% ± 0.02%, respectively, compared to 0.58% ± 0.01%, 0.58% ± 0.01%, and 0.45% ± 0.04% for C-RT. A similar trend was observed when the template bases were A and C, where C-RT consistently demonstrated higher readthrough efficiencies. For A-A and A-C mismatches, B-RT exhibited readthrough efficiencies of 0.33% ± 0.02% and 2.68% ± 0.09%, respectively, compared to 0.56% ± 0.03% and 4.09% ± 0.18% for C-RT. Likewise, for C-A and C-C mismatches, B-RT showed values of 0.30% ± 0.07% and 0.22% ± 0.01%, whereas C-RT exhibited 0.54% ± 0.05% and 0.46% ± 0.07%, respectively. In contrast, when the template base was G, a significant difference between subtypes was observed for only one mismatch combination. Specifically, the G-T mismatch yielded readthrough efficiencies of 0.29% ± 0.04% for B-RT and 0.53% ± 0.05% for C-RT. On average, the canonical HIV-1C RT exhibited a 1.6-fold higher mismatch-extension efficiency than the canonical G359 HIV-1B RT. Introducing threonine into B-RT did not produce statistically significant differences across any template-primer combinations. Conversely, substitution of glycine for threonine at position 359 in subtype C RT resulted in a reduction in mismatch extension efficiency in a subset of template-primer combinations, specifically A-C, C-A, C-C, and T-C. For the A-C mismatch, readthrough efficiency declined from 4.09% ± 0.18% in wild-type C-RT to 2.47% ± 0.41% in the G359 variant. Similarly, for the C-A, C-C, and T-C mismatches, efficiencies decreased from 0.54% ± 0.05% to 0.19% ± 0.04%, 0.46% ± 0.07% to 0.30% ± 0.04%, and 0.58% ± 0.01% to 0.37% ± 0.06%, respectively. Notably, no consistent reduction was observed across other mismatch combinations, indicating that the effect of the G359 substitution is selective rather than global. Conversely, substituting glycine for threonine in C-RT reduced mismatch extension efficiency in only four cases (A-C, C-A, C-C, and U-C). Serine substitutions produced patterns broadly consistent with the respective wild-type residues within each subtype (Fig. 6B). Serine substitutions produced patterns broadly consistent with the respective wild-type residues within each subtype (Fig. 6B).

Collectively, these results indicate that HIV-1C RT possesses an enhanced intrinsic capacity to extend mismatched nucleotides relative to HIV-1B RT. This property, together with MHD-mediated annealing, provides a mechanistic explanation for the higher frequency and greater diversity of sequence motif duplications observed in HIV-1C.

## DISCUSSION

### HIV-1C RT mediates enhanced strand transfer and mismatch extension events

HIV-1C exhibits a substantially higher frequency of sequence motif duplications at specific genomic hotspots, particularly in the LTR and p6-Gag regions (4, 6, 7, 9, 12), where duplications arise through nonhomologous recombination (3, 29). These observations have raised the possibility that subtype-specific differences in recombination or RT function may underlie this bias. However, previous studies (21, 30), together with our current data, indicate that overall recombination rates are broadly comparable across subtypes, suggesting that factors beyond bulk recombination frequency must explain the elevated duplication propensity of HIV-1C.

To our knowledge, no study has directly compared the strand-switching capacity of RTs from different HIV-1 subtypes. Several prior analyses relied on the cell culture-based EGFP complementation assay to measure recombination frequency (22, 23, 31, 32). However, this approach lacks the resolution to detect subtle differences in RT strand switching and assesses recombination over only a ~600 bp region, assuming uniform recombination across the genome. Such an assumption may overlook recombination hotspots and RNA secondary structures. Our study shares this limitation, as additional reporter systems were not evaluated. Nevertheless, because sequence duplication arises from nonhomologous recombination, estimated to occur 10–100 times less frequently than homologous recombination (3), the strand-transfer assay provides a relevant surrogate. By quantifying cDNA synthesis on the acceptor strand after a forced transfer, it models conditions that approximate duplication events.

Nonhomologous recombination poses two principal challenges. First, the nascent cDNA-RNA intermediate is inherently unstable due to minimal complementarity between the 3′ end of the DNA and short microhomology regions. We previously proposed that the formation of a transient DNA loop constitutes a critical intermediate in duplication (24). Second, terminal mismatches are common, occurring in approximately three out of four events, and have been shown to promote strand transfer (13–15). For duplication to be successfully completed, these mismatches must be extended rather than resolved by an additional strand switch, requiring RT to tolerate mismatches while maintaining processive DNA synthesis.

Our data suggest that HIV-1C RT is uniquely adapted to meet these challenges. The presence of threonine at position 359 in the connection domain may stabilize the looped intermediate, potentially through an additional hydrogen bond with the nascent DNA. In parallel, HIV-1C RT exhibits enhanced mismatch-extension capacity (Fig. 6B), enabling continued synthesis across imperfectly paired termini. Together, these properties likely reduce the probability of abortive strand transfers and promote completion of duplication events at hotspots (Fig. 1C). Notably, the observed increase in mismatch extension (~1.6-fold) is modest, consistent with the need to preserve replication fidelity and avoid excessive mutagenesis.

Importantly, RNA secondary structures are known to influence reverse transcription by inducing RT pausing and promoting strand transfer (2, 32, 33). In principle, such pausing and switching can occur anywhere along the genome where suitable structural elements exist, and therefore nonhomologous recombination, and consequently sequence duplication, can, in theory, arise at any location. Consistent with this, we observe sporadic duplication events distributed across the genome (Fig. 1A), indicating that the underlying mechanism is not restricted to specific loci.

At the same time, local sequence context and RNA structural propensity may differ between subtypes and could contribute to subtype-specific duplication patterns. A notable example is the HIV-1C LTR, where the presence of an additional NF-κB (C-κB) site alters the local architecture. This modification enables duplication of the upstream H-κB site, a pattern not observed in other subtypes, which instead exhibit duplications in alternative regions in the LTR (4). Thus, subtype-specific sequence features may influence where duplications occur.

However, our strand-transfer assay used the same RNA template for both HIV-1B and HIV-1C RTs. Under these controlled conditions, RNA secondary structures are constant, and the observed differences in strand transfer and extension efficiency can therefore be attributed to intrinsic properties of the RTs themselves. These findings suggest that, while RNA structure likely shapes the distribution of duplication hotspots, subtype-specific biochemical differences in RT contribute to the overall increased frequency of duplication in HIV-1C.

In summary, enhanced strand transfer and improved mismatch extension provide a mechanistic explanation for the elevated frequency of sequence motif duplications in HIV-1C. Further work is needed to define the contribution of additional signature residues to the distinct biochemical properties of HIV-1C RT.

### Signature amino acid residues may modulate the overall performance of the RT

Bioinformatic comparison of RT sequences across HIV-1 subtypes identified several residues unique to HIV-1C (Table 1). We focused on the non-conservative substitution of glycine with threonine at position 359 in the connection domain. Given its proximity to the nascent DNA, threonine could form an additional hydrogen bond with the growing strand. Although the absence of a resolved HIV-1C RT crystal structure limits direct confirmation, two lines of evidence support this hypothesis. First, replacing T359 with glycine, which lacks a hydroxyl group, markedly increased RT activity (Fig. 3B), whereas substitution with serine, capable of forming a weaker hydrogen bond, produced only a modest increase. Second, because HIV-1B and HIV-1C RTs share 88%–93% sequence identity, structural modeling based on the HIV-1B RT structure reliably predicts hydrogen bond formation at position 359 (Fig. 2C).

Consistent with prior reports (16), we observed comparable specific activities for wild-type HIV-1B and HIV-1C RTs (Fig. 3B). However, substituting glycine for threonine at position 359 increased C-RT activity several-fold, suggesting that the threonine-mediated hydrogen bond constrains polymerase activity. Given the tightly coordinated nature of reverse transcription, excessive RT activity could disrupt replication kinetics. Supporting this interpretation, the threonine-containing variant showed superior p24 production relative to the glycine variant (Fig. 3D).

Although our analysis centers on T359, other HIV-1C-specific residues likely contribute to RT function. Notably, C-RT harboring glycine at position 359 displays approximately sixfold higher polymerase activity than B-RT, implying additional cooperative effects. Thus, T359 may act in a compensatory manner to maintain optimal polymerization efficiency. We identified five additional subtype-specific residues, including A36 and T48 near the dNTP-binding pocket in the fingers domain. Because this domain interacts extensively with viral RNA (34, 35), variation at these positions may further influence strand transfer and mismatch extension in HIV-1C RT.

### Selection of duplication hotspots and evolutionary consequences

Darwinian selection shapes HIV-1 evolution by favoring fitter variants. Although sequence duplications in HIV-1C can arise across the genome, they are concentrated at discrete hotspots. Given the link between recombination and duplication, these hotspots are expected to overlap. Database analyses identify four major regions: LTR, p6, env, and nef. Functional studies show that LTR and p6 duplications confer replication advantages (4–6), indicating positive selection. Duplications in env and nef may similarly enhance fitness, possibly via immune evasion or altered pathogenicity, but remain unvalidated. In contrast, duplications elsewhere are likely deleterious and removed by purifying selection. Thus, while HIV-1C RT may generate duplications broadly, only advantageous variants persist.

LTR duplications may have broader clinical implications. The LTR regulates gene expression and latency and is highly sensitive to structural changes. Additional NF-κB sites increase transcription (4), whereas RBE-III duplication appears to reduce latency reactivation. Our unpublished data suggest that dual RBE-III variants are refractory to latency-reversing agents, potentially promoting more stable reservoirs (D. Bhange, A. Panchapakesan, S. Mishra, S. Singh, D. Parihar, C. Saini, M. Shanmugam, H. Buch, J. Singh, R. Manna, M. Sharma, S. Suresh, G. Selvamurthi, N. Nala, S. N. Byrareddy, S. P. Maurya, M. Dias, B. K. Das, K. G. Murugavel, A. K. Srikrishnan, T. K. Kundu, and U. Ranga, unpublished data).

Together, these findings indicate that the high duplication frequency in HIV-1C reflects adaptive evolution rather than stochastic error. By enhancing fitness, transmission, and persistence under therapy, this phenotype has important implications for global HIV control, particularly given the prevalence of HIV-1C.

### Limitations of the study and future directions

The distinctive nature of sequence-motif duplications in HIV-1 presents significant experimental challenges. These events arise from rare, multi-step processes during reverse transcription involving nonhomologous strand transfer and template switching. Given recombination frequencies of ~0.03%–0.05% per residue across the 9.5 kb genome (36), and the fact that nonhomologous recombination occurs 100- to 1,000-fold less frequently than homologous recombination (3), the estimated duplication frequency at any specific site is extremely low (~0.000015%–0.0025%). This is further compounded by the observation that ~95% of proviruses are defective due to large deletions or lethal mutations (37, 38), making direct detection of duplication events in controlled systems highly impractical.

Consistent with these constraints, prolonged viral culture experiments failed to yield detectable duplications (data not shown). Serial passaging of the HIV-1C Indie clone for over 4 months did not generate variants such as the 4 NF-κB LTR. *In vitro* systems are further limited by the predominance of homologous recombination, which suppresses nonhomologous outcomes. These limitations necessitated the use of indirect approaches, including comparative sequence analysis, biochemical assays, and computational inference.

Accordingly, while our data demonstrate enhanced strand transfer and mismatch extension by HIV-1C RT, we cannot directly link these properties to duplication events *in vitro* or *in vivo*. Additional factors, including nucleocapsid activity, RNA secondary structure, and sequence context, likely contribute and remain to be systematically examined. Nevertheless, the observed biochemical differences support a model in which subtype-specific RT properties increase duplication probability under physiological conditions. Importantly, inter-subtype chimeric RTs exhibit reduced enzymatic efficiency (17, 39, 40), underscoring the functional importance of subtype-specific residues. Collectively, our findings provide mechanistic insight into this phenotype and identify candidate residues that may contribute to duplication propensity, warranting further investigation.

## MATERIALS AND METHODS

### Sequence and structural analysis

HIV-1 sequences were retrieved from the Los Alamos National Laboratory HIV Sequence Database and aligned using Clustal Omega with default parameters. Multiple sequence alignments were examined and curated using BioEdit version 7.2.5. Structural visualization and modeling of the RT were performed using PyMOL version 2.4.1. A high-resolution crystal structure of HIV-1B RT (PDB ID: 5J2M [41]) was used as the template. HIV-1C RT–specific signature amino acid residues were introduced into this structure using the mutagenesis tool in PyMOL, followed by local energy minimization to optimize side-chain conformations. The bond length between the T359 residue and the DNA phosphate (PO₄) group was measured using the built-in distance calculation tools in the software.

### Construction of plasmids

In all experiments, we used the NL4-3 (GenBank accession number AF324493.2) and Indie-C1 (AB023804.1) molecular clones as representatives of HIV-1B and −1C, respectively. The NL4-3 and NL4-3-ΔEnv-EGFP molecular clones were obtained through the NIH-AIDS Reagent Program of the Division of AIDS, NIAID, NIH (Cat. Nos: ARP-2852 and ARP-11100, contributed by Dr. M. Martin, Dr. Haili Zhang, Dr. Yan Zhou, and Dr. Robert Siliciano, respectively). The Indie-C1 molecular clone was a kind gift from Dr. Masashi Tatsumi (International Medical Center of Japan, Tokyo). The Indie-ΔEnv-EGFP reporter vector was generated in our laboratory. Prof. Vinayaka Prasad (Albert Einstein College of Medicine, New York) kindly provided the RT expression vectors p6HRT and p6HRT-p51, originally developed by Stuart LeGrice and Fiona Gruninger-Leitch (42). All plasmid constructs were cloned into *E. coli* XL-10 Gold cells, grown at 30°C overnight. All primers were synthesized by Sigma-Aldrich, Bengaluru, India, and enzymes for cloning and PCR were procured from New England Biolabs (USA). Additional reagents used in specific assays are indicated where relevant.

Panels of full-length infectious molecular clones were generated for both NL4-3 and Indie-C1 backbones, differing by a single amino acid at position 359 of RT (glycine, threonine, serine). Site-directed mutagenesis was performed using overlap-extension PCR with Phusion DNA polymerase and primer sets listed in File S1. Mutant fragments were assembled using outer primer pairs, digested with AgeI/EcoRI (for NL4-3) or PflMI (for Indie-C1), and substituted into the parental backbones via restriction enzyme–mediated fragment exchange. Recombinant clones were identified by restriction digestion and confirmed by Sanger sequencing. The resulting constructs were designated according to the amino acid at position 359 (e.g., Indie–359G).

The RT expression vectors p6HRT (p66 subunit) and p6HRT-p51 (p51 subunit) contain an N-terminal 6×His tag under the control of the lac promoter. The corresponding NL4-3 or Indie RT variants were cloned into the p6HRT and p6HRT-p51 backbones using BamHI/SalI and BamHI/HindIII restriction sites, respectively. A unique restriction site was incorporated into the reverse primer beyond the stop codon to serve as a surrogate identifier for the amino acid at position 359. For the Indie constructs, an additional SacI site was introduced for differentiation from the NL4-3 panel.

Reporter constructs were derived from NL4-3 ΔEnv EGFP and Indie ΔEnv EGFP backbones. The RT region was modified by swapping restriction fragments (AgeI/EcoRI for NL4-3 and PflMI for Indie) from the corresponding full-length RT mutant clones. Each of the eight intermediate RT variant reporters was then used to generate two EGFP frameshift mutants, carrying insertions at amino acid positions 4 or 204 of the GFP open reading frame. Mutations were introduced by direct or overlap PCR and cloned directionally using EcoRI/NheI (for NL4-3) or SphI/StuI (for Indie) restriction sites. Recombinant clones were verified by restriction digestion and confirmed by Sanger sequencing.

### Expression and purification of recombinant RTs

Recombinant HIV-1 RT expression constructs (p6HRT and p6HRT-p51) were transformed separately into *E. coli* M15-competent cells (42). Protein expression was induced with 1 mM IPTG at an OD₆₀₀ of 0.4 and continued for 5 h at 37°C with shaking. Cells were harvested, resuspended in 50 mM sodium phosphate buffer (pH 8.0) containing 150 mM NaCl and 1 mM PMSF, and lysed by sonication (VCX-130, Sonics and Materials Inc., USA). The clarified lysate (12,000 × *g*, 10 min) was incubated overnight at 4°C with Ni–NTA resin (0.5 mL resin/200 mL lysate; G-Biosciences, USA).

The lysate was then washed with 50 mM sodium phosphate (pH 8.0), 20 mM imidazole, 150 mM NaCl, 1 mM PMSF, and the bound RT was eluted with 250 mM imidazole in the same buffer. Eluted fractions were pooled and dialyzed overnight against 50 mM Tris-Cl (pH 7.0), 25 mM NaCl, 1 mM EDTA, and 20% glycerol. Further purification was achieved using DEAE-Sepharose ion-exchange chromatography, collecting the flow-through fraction containing RT. Final enzyme preparations were dialyzed into 50 mM Tris-Cl (pH 8.0), 150 mM NaCl, 1 mM DTT, 50% glycerol (43), concentrated using Amicon concentrators (Merck-Millipore, USA), and stored in aliquots at −20°C until use.

### Determination of RT activity

RT activity was assayed using a poly(U)/oligo(dA)₍₂₀₎ template–primer system as described previously (44), with minor modifications. Briefly, 4 µg polyuridylic acid (Sigma-Aldrich, USA) was annealed with 1 µg (~80 pmol) oligo(dA)₍₂₀₎ primer by heating at 95°C, followed by rapid cooling on ice. The template–primer complex was incubated in a 20 µL reaction containing 25 mM Tris-Cl (pH 8.0), 3 mM MgCl₂, 100 mM KCl, and 250 µM dATP (GeNei Labs, India), supplemented with 1 µCi [α-³²P]-dATP (Board of Radiation & Isotope Technologies, India).

Reactions were initiated with varying enzyme concentrations (0.1–1 µL) and incubated at 37°C for 10 min, then terminated with 0.5 M EDTA. Aliquots were spotted on Hybond-N^+^ nylon membranes (Amersham Lifesciences, USA), washed to remove unincorporated nucleotides, ethanol-rinsed, and air-dried. Radioactivity incorporated into cDNA was quantified by liquid scintillation counting (MicroBeta², PerkinElmer, USA) using Ultima Gold XR scintillation fluid.

Specific activity was calculated as the amount of enzyme incorporating 1 pmol [α-³²P]-dATP in 10 min at 37°C per µg of protein. For rate determination, reactions were performed as above with 1 µL enzyme, and nucleotide incorporation was measured at 5-min intervals.

### Cell culture, virus production, and titration

T-cell lines CEM-CCR5, Jurkat, Jurkat-CCR5, and epithelial cell lines HEK 293T and TZM-bl were maintained in RPMI 1640 or DMEM, respectively (Sigma-Aldrich, USA), supplemented with 10% FBS (Life Technologies, India), 2 mM glutamine, and 100 µg/mL each of penicillin G and streptomycin (Sigma-Aldrich, USA). All cell cultures were incubated at 37°C in 5% CO₂. Peripheral blood mononuclear cells (PBMCs) were isolated from fresh donor blood by density gradient centrifugation. CD8^+^ cells were depleted using the RosetteSep Human CD8 Depletion Cocktail (STEMCELL Technologies, Canada). PBMCs were activated for 72 h in RPMI 1640 containing 20 U/mL IL-2, 5 µg/mL phytohemagglutinin-P (PHA-P), and the supplements described above. Prior to infection, cells were maintained without PHA-P. Infectious HIV-1 viral stocks were generated by transient transfection of HEK 293T cells. Approximately 3 × 10⁶ cells were seeded in 100 mm dishes and transfected with 20 µg of molecular clone DNA and 30 ng of pCMV-TdTomato (transfection control) using the calcium phosphate method (45). For recombination assays, pseudotyped viral variants were produced in six-well plates by transfecting 0.3 × 10⁶ HEK 293T cells with a mixture containing 3 µg of reporter vector, 1 µg of pCMV-VSV-G, and 10 ng of pCMV-TdTomato. Viruses copackaging two RNA genomes, each harboring complementary debilitating mutations in the GFP ORF, were produced by cotransfecting equal amounts (1.5 µg each) of the corresponding plasmids. Following transfection, the culture medium was replaced after 6 h, and supernatants were harvested at 48 h post-transfection, filtered through 0.22 µm syringe filters, and stored in aliquots at −80°C. Viral yield was quantified by p24 ELISA (4th-Generation HIV-1 p24 Antigen ELISA Kit, J. Mitra & Co. Pvt. Ltd., India). Functional infectivity was assessed by infecting TZM-bl cells with serial dilutions of the viral stock. Briefly, 1 × 10⁴ cells were seeded per well in 96-well plates and infected in the presence of 10 µg/mL DEAE-dextran. After 8 h, the inoculum was replaced with complete DMEM, and cells were incubated for 48 h at 37°C and 5% CO₂. Infection efficiency was quantified by measuring firefly luciferase activity in cell lysates using the Luciferase Assay System (Promega, USA) on a Varioskan Lux multimode reader (Thermo Fisher Scientific, USA).

### Recombination assay

CEM-CCR5 or Jurkat cells (0.3 × 10⁶) were infected with 10 ng p24 equivalent of either NL4-3 or Indie ΔEnv EGFP variant viruses in 2 mL RPMI medium containing 10 µg/mL DEAE-dextran in six-well plates. The culture medium was replaced with fresh RPMI 6 h post-infection. Twenty-four hours before analysis, cells were activated with a cocktail containing 10 ng/mL TNF-α (Miltenyi Biotec, USA), 5 ng/mL PMA, and 5 mM HMBA (Sigma-Aldrich, USA). Cells were harvested by centrifugation at 500 × *g*, washed twice with PBS, and resuspended in 250 µL PBS with 2% FBS. Flow cytometric analysis was performed using a BD FACS ARIA III (Becton, Dickinson and Company, USA) to quantify GFP expression, which served as a measure of recombination frequency.

### Strand-transfer assay

Two regions of the Indie genome (HXB2 coordinates 256–462 and 473–603) were amplified using forward primers carrying a T7 promoter (File S1) to enable *in vitro* transcription. The second fragment contained a 20 bp sequence at its 3′ end homologous to the 5′ end of the first fragment, allowing potential strand transfer during reverse transcription. RNAs were synthesized and purified, mixed at an equimolar ratio, and reverse transcribed using 1 Unit of RT and the antisense primer N4864, which anneals to RNA-1, under the same conditions. Two microliters of the cDNA product was diluted 1,000-fold and used as templates for two qPCRs: one with primers N4863/N4864 to detect the RNA-1 template, and another with N4865/N4864 to detect strand-transfer products.

### Mismatch extension assay

A 331-nt fragment of the HIV-1C genome (HXB2 coordinates: 1583–1914) was PCR-amplified using primers containing the T7 promoter sequence to enable *in vitro* transcription. The resulting RNA served as the template, with a defined four-base stretch (ATGC) used as the initiation site for polymerization (Fig. 6A). Sixteen reverse primers, grouped by an initiation base, were designed so that each group initiated synthesis at one of the four template bases. Within each group, primers were identical except for the 3′ terminal nucleotide, yielding all sixteen possible base pair combinations at the primer–template junction: four correct (A–T, T–A, G–C, C–G) and twelve mismatched pairs. Three independent sets of the sixteen primers were synthesized, each carrying a unique 8 bp barcode at the 5′ end to enable multiplexed next-generation sequencing (NGS). Primer sequences are listed in File S1. Annealing was performed by mixing 2 µg of template with 100 pmol of the appropriate primer, and the extension reactions were carried out using the six recombinant RT variants under identical conditions. Reaction products were treated with RNase H, purified, and subjected to second-strand synthesis using Taq DNA polymerase. Products were then purified, and equal reaction volumes were pooled for end repair and library preparation, followed by paired-end Illumina sequencing on a NextSeq 2000 platform. Sequencing data were analyzed using a custom Python pipeline.

## Data Availability

All custom Python scripts used for data analysis in this study have been deposited at the laboratory’s GitHub page (https://github.com/hivaidslab). Additional information and materials are available from the corresponding author upon request.

## SUPPLEMENTAL MATERIAL

File S1 jvi.00526-26-s0001.xlsx Primer sequences used in the study.

Supplemental tables jvi.00526-26-s0002.pdf Tables S1 and S2.

ASM does not own the copyrights to Supplemental Material that may be linked to, or accessed through, an article. The authors have granted ASM a non-exclusive, world-wide license to publish the Supplemental Material files. Please contact the corresponding author directly for reuse.

## References

[1] Hu W-S, Hughes SH. 2012. HIV-1 reverse transcription. Cold Spring Harb Perspect Med 2:a006882. doi:10.1101/cshperspect.a006882 23028129 PMC3475395

[2] Onafuwa-Nuga A, Telesnitsky A. 2009. The remarkable frequency of Human immunodeficiency virus type 1 genetic recombination. Microbiol Mol Biol Rev 73:451–480. doi:10.1128/MMBR.00012-09 19721086 PMC2738136

[3] Zhang J, Temin HM. 1993. Rate and mechanism of nonhomologous recombination during a single cycle of retroviral replication. Science 259:234–238. doi:10.1126/science.8421784 8421784

[4] Bachu M, Yalla S, Asokan M, Verma A, Neogi U, Sharma S, Murali RV, Mukthey AB, Bhatt R, Chatterjee S, Rajan RE, Cheedarla N, Yadavalli VS, Mahadevan A, Shankar SK, Rajagopalan N, Shet A, Saravanan S, Balakrishnan P, Solomon S, Vajpayee M, Satish KS, Kundu TK, Jeang K-T, Ranga U. 2012. Multiple NF-κB sites in HIV-1 subtype C long terminal repeat confer superior magnitude of transcription and thereby the enhanced viral predominance. J Biol Chem 287:44714–44735. doi:10.1074/jbc.M112.397158 23132857 PMC3531786

[5] Martins AN, Waheed AA, Ablan SD, Huang W, Newton A, Petropoulos CJ, Brindeiro RDM, Freed EO. 2016. Elucidation of the molecular mechanism driving duplication of the HIV-1 PTAP late domain. J Virol 90:768–779. doi:10.1128/JVI.01640-15 26512081 PMC4702686

[6] Sharma S, Arunachalam PS, Menon M, Ragupathy V, Satya RV, Jebaraj J, Aralaguppe SG, Rao C, Pal S, Saravanan S, Murugavel KG, Balakrishnan P, Solomon S, Hewlett I, Ranga U. 2018. PTAP motif duplication in the p6 Gag protein confers a replication advantage on HIV-1 subtype C. J Biol Chem 293:11687–11708. doi:10.1074/jbc.M117.815829 29773649 PMC6066301

[7] Sharma S, Aralaguppe SG, Abrahams M-R, Williamson C, Gray C, Balakrishnan P, Saravanan S, Murugavel KG, Solomon S, Ranga U. 2017. The PTAP sequence duplication in HIV-1 subtype C Gag p6 in drug-naive subjects of India and South Africa. BMC Infect Dis 17:95. doi:10.1186/s12879-017-2184-4 28118816 PMC5259826

[8] Brindeiro PA, Brindeiro RM, Mortensen C, Hertogs K, De Vroey V, Rubini NPM, Sion FS, De Sá CAM, Machado DM, Succi RCM, Tanuri A. 2002. Testing genotypic and phenotypic resistance in Human immunodeficiency virus type 1 isolates of clade B and other clades from children failing antiretroviral therapy. J Clin Microbiol 40:4512–4519. doi:10.1128/JCM.40.12.4512-4519.2002 12454144 PMC154623

[9] Martins AN, Arruda MB, Pires AF, Tanuri A, Brindeiro RM. 2011. Accumulation of P(T/S)AP late domain duplications in HIV type 1 subtypes B, C, and F derived from individuals failing ARV therapy and ARV drug-naive patients. AIDS Res Hum Retroviruses 27:687–692. doi:10.1089/aid.2010.0282 21083435

[10] Peters S, Muñoz M, Yerly S, Sanchez-Merino V, Lopez-Galindez C, Perrin L, Larder B, Cmarko D, Fakan S, Meylan P, Telenti A. 2001. Resistance to nucleoside analog reverse transcriptase inhibitors mediated by Human immunodeficiency virus type 1 p6 protein. J Virol 75:9644–9653. doi:10.1128/JVI.75.20.9644-9653.2001 11559796 PMC114535

[11] Tamiya S, Mardy S, Kavlick MF, Yoshimura K, Mistuya H. 2004. Amino acid insertions near Gag cleavage sites restore the otherwise compromised replication of Human immunodeficiency virus type 1 variants resistant to protease inhibitors. J Virol 78:12030–12040. doi:10.1128/JVI.78.21.12030-12040.2004 15479842 PMC523239

[12] Bachu M, Mukthey AB, Murali RV, Cheedarla N, Mahadevan A, Shankar SK, Satish KS, Kundu TK, Ranga U. 2012. Sequence insertions in the HIV type 1 subtype C viral promoter predominantly generate an additional NF-κB binding site. AIDS Res Hum Retroviruses 28:1362–1368. doi:10.1089/aid.2011.0388 22332607 PMC3448112

[13] Chin MPS, Chen J, Nikolaitchik OA, Hu W-S. 2007. Molecular determinants of HIV-1 intersubtype recombination potential. Virology 363:437–446. doi:10.1016/j.virol.2007.01.034 17336363

[14] Palaniappan C, Wisniewski M, Wu W, Fay PJ, Bambara RA. 1996. Misincorporation by HIV-1 reverse transcriptase promotes recombination via strand transfer synthesis. J Biol Chem 271:22331–22338. doi:10.1074/jbc.271.37.22331 8798393

[15] Schlub TE, Grimm AJ, Smyth RP, Cromer D, Chopra A, Mallal S, Venturi V, Waugh C, Mak J, Davenport MP. 2014. Fifteen to twenty percent of HIV Substitution Mutations Are Associated with Recombination. J Virol 88:3837–3849. doi:10.1128/JVI.03136-13 24453357 PMC3993552

[16] Xu H-T, Quan Y, Asahchop E, Oliveira M, Moisi D, Wainberg MA. 2010. Comparative biochemical analysis of recombinant reverse transcriptase enzymes of HIV-1 subtype B and subtype C. Retrovirology 7:80. doi:10.1186/1742-4690-7-80 20929562 PMC2959035

[17] Iordanskiy S, Waltke M, Feng Y, Wood C. 2010. Subtype-associated differences in HIV-1 reverse transcription affect the viral replication. Retrovirology 7:85. doi:10.1186/1742-4690-7-85 20939905 PMC2964588

[18] Korber B, Myers G. 1992. Signature pattern analysis: a method for assessing viral sequence relatedness. AIDS Res Hum Retrovir 8:1549–1560. doi:10.1089/aid.1992.8.1549 1457200

[19] Abramson J, Adler J, Dunger J, Evans R, Green T, Pritzel A, Ronneberger O, Willmore L, Ballard AJ, Bambrick J, et al.. 2024. Accurate structure prediction of biomolecular interactions with AlphaFold 3. Nature 630:493–500. doi:10.1038/s41586-024-07487-w 38718835 PMC11168924

[20] Murray JM, Kelleher AD, Cooper DA. 2011. Timing of the components of the HIV life cycle in productively infected CD4+ T cells in a population of HIV-infected individuals. J Virol 85:10798–10805. doi:10.1128/JVI.05095-11 21835801 PMC3187481

[21] Chin MPS, Rhodes TD, Chen J, Fu W, Hu W-S. 2005. Identification of a major restriction in HIV-1 intersubtype recombination. Proc Natl Acad Sci USA 102:9002–9007. doi:10.1073/pnas.0502522102 15956186 PMC1157039

[22] Levy DN, Aldrovandi GM, Kutsch O, Shaw GM. 2004. Dynamics of HIV-1 recombination in its natural target cells. Proc Natl Acad Sci USA 101:4204–4209. doi:10.1073/pnas.0306764101 15010526 PMC384719

[23] Rhodes TD, Nikolaitchik O, Chen J, Powell D, Hu W-S. 2005. Genetic recombination of Human immunodeficiency virus type 1 in one round of viral replication: effects of genetic distance, target cells, accessory genes, and lack of high negative interference in crossover events. J Virol 79:1666–1677. doi:10.1128/JVI.79.3.1666-1677.2005 15650192 PMC544095

[24] Panchapakesan A, Ranga U. 2025. A model of non-homologous recombination mediated by HIV-1 reverse transcriptase explaining sequence motif duplications that confer a replication fitness advantage. Viruses 17:680. doi:10.3390/v17050680 40431692 PMC12115494

[25] Perrino FW, Preston BD, Sandell LL, Loeb LA. 1989. Extension of mismatched 3’ termini of DNA is a major determinant of the infidelity of Human immunodeficiency virus type 1 reverse transcriptase. Proc Natl Acad Sci USA 86:8343–8347. doi:10.1073/pnas.86.21.8343 2479023 PMC298277

[26] Bakhanashvili M, Hizi A. 1996. The interaction of the reverse transcriptase of Human immunodeficiency virus type 1 with 3’-terminally mispaired DNA. Arch Biochem Biophys 334:89–96. doi:10.1006/abbi.1996.0433 8837743

[27] Menéndez-Arias L. 1998. Studies on the effects of truncating alpha-helix E’ of p66 Human immunodeficiency virus type 1 reverse transcriptase on template-primer binding and fidelity of DNA synthesis. Biochemistry 37:16636–16644. doi:10.1021/bi981830g 9843431

[28] Kharytonchyk S, King SR, Ndongmo CB, Stilger KL, An W, Telesnitsky A. 2016. Resolution of specific nucleotide mismatches by wild-type and AZT-resistant reverse transcriptases during HIV-1 replication. J Mol Biol 428:2275–2288. doi:10.1016/j.jmb.2016.04.005 27075671 PMC4884515

[29] Yin PD, Pathak VK, Rowan AE, Teufel RJ, Hu WS. 1997. Utilization of nonhomologous minus-strand DNA transfer to generate recombinant retroviruses. J Virol 71:2487–2494. doi:10.1128/JVI.71.3.2487-2494.1997 9032388 PMC191361

[30] Galli A, Kearney M, Nikolaitchik OA, Yu S, Chin MPS, Maldarelli F, Coffin JM, Pathak VK, Hu W-S. 2010. Patterns of Human immunodeficiency virus type 1 recombination ex vivo provide evidence for coadaptation of distant sites, resulting in purifying selection for intersubtype recombinants during replication. J Virol 84:7651–7661. doi:10.1128/JVI.00276-10 20504919 PMC2897624

[31] Motomura K, Chen J, Hu W-S. 2008. Genetic recombination between Human immunodeficiency virus type 1 (HIV-1) and HIV-2, two distinct human Lentiviruses. J Virol 82:1923–1933. doi:10.1128/JVI.01937-07 18057256 PMC2258735

[32] Nikolaitchik OA, Galli A, Moore MD, Pathak VK, Hu W-S. 2011. Multiple barriers to recombination between divergent HIV-1 variants revealed by a dual-marker recombination assay. J Mol Biol 407:521–531. doi:10.1016/j.jmb.2011.01.052 21295586 PMC3065980

[33] Simon-Loriere E, Martin DP, Weeks KM, Negroni M. 2010. RNA structures facilitate recombination-mediated gene swapping in HIV-1. J Virol 84:12675–12682. doi:10.1128/JVI.01302-10 20881047 PMC3004330

[34] Patel PH, Loeb LA. 2001. Getting a grip on how DNA polymerases function. Nat Struct Biol 8:656–659. doi:10.1038/90344 11473246

[35] Warrilow D, Tachedjian G, Harrich D. 2009. Maturation of the HIV reverse transcription complex: putting the jigsaw together. Rev Med Virol 19:324–337. doi:10.1002/rmv.627 19750561

[36] Zhuang J, Jetzt AE, Sun G, Yu H, Klarmann G, Ron Y, Preston BD, Dougherty JP. 2002. Human immunodeficiency virus type 1 recombination: rate, fidelity, and putative hot spots. J Virol 76:11273–11282. doi:10.1128/jvi.76.22.11273-11282.2002 12388687 PMC136766

[37] Imamichi H, Smith M, Adelsberger JW, Izumi T, Scrimieri F, Sherman BT, Rehm CA, Imamichi T, Pau A, Catalfamo M, Fauci AS, Lane HC. 2020. Defective HIV-1 proviruses produce viral proteins. Proc Natl Acad Sci USA 117:3704–3710. doi:10.1073/pnas.1917876117 32029589 PMC7035625

[38] Pollack RA, Jones RB, Pertea M, Bruner KM, Martin AR, Thomas AS, Capoferri AA, Beg SA, Huang S-H, Karandish S, Hao H, Halper-Stromberg E, Yong PC, Kovacs C, Benko E, Siliciano RF, Ho Y-C. 2017. Defective HIV-1 proviruses are expressed and can be recognized by cytotoxic T lymphocytes, which shape the proviral landscape. Cell Host Microbe 21:494–506. doi:10.1016/j.chom.2017.03.008 28407485 PMC5433942

[39] Brenner BG, Oliveira M, Doualla-Bell F, Moisi DD, Ntemgwa M, Frankel F, Essex M, Wainberg MA. 2006. HIV-1 subtype C viruses rapidly develop K65R resistance to tenofovir in cell culture. AIDS 20:F9–13. doi:10.1097/01.aids.0000232228.88511.0b 16816549

[40] Nagata S, Imai J, Makino G, Tomita M, Kanai A. 2017. Evolutionary analysis of HIV-1 pol proteins reveals representative residues for viral subtype differentiation. Front Microbiol 8:2151. doi:10.3389/fmicb.2017.02151 29163435 PMC5666293

[41] Salie ZL, Kirby KA, Michailidis E, Marchand B, Singh K, Rohan LC, Kodama EN, Mitsuya H, Parniak MA, Sarafianos SG. 2016. Structural basis of HIV inhibition by translocation-defective RT inhibitor 4’-ethynyl-2-fluoro-2’-deoxyadenosine (EFdA). Proc Natl Acad Sci USA 113:9274–9279. doi:10.1073/pnas.1605223113 27489345 PMC4995989

[42] Le Grice SFJ, Grüninger‐leitch F. 1990. Rapid purification of homodimer and heterodimer HIV‐1 reverse transcriptase by metal chelate affinity chromatography. Eur J Biochem 187:307–314. doi:10.1111/j.1432-1033.1990.tb15306.x 1688798

[43] Stahlhut MW, Olsen DB. 1996. Expression and purification of retroviral HIV-1 reverse transcriptase. Methods Enzymol 275:122–133. doi:10.1016/s0076-6879(96)75010-3 9026635

[44] Le Grice SF, Cameron CE, Benkovic SJ. 1995. Purification and characterization of Human immunodeficiency virus type 1 reverse transcriptase. Methods Enzymol 262:130–144. doi:10.1016/0076-6879(95)62015-x 8594344

[45] Kingston RE, Chen CA, Rose JK. 2003. Calcium phosphate transfection. CP Molecular Biology 63:9. doi:10.1002/0471142727.mb0901s63 18265332

