# Supplementary material for: Enhanced strand transfer and mismatch extension by HIV-1C reverse transcriptase promote sequence motif duplication
Source: J Virol. 2026 Jun 4;100(6):e00526-26. doi: 10.1128/jvi.00526-26 (PMC13288777; doi:10.1128/jvi.00526-26)
Supplement: Supplemental tables — Tables S1 and S2. [file jvi.00526-26-s0002.pdf]

		Amino Acid																			
**Position**	**Subtype**	**A**	**C**	**D**	**E**	**F**	**G**	**H**	**I**	**K**	**L**	**M**	**N**	**P**	**Q**	**R**	**S**	**T**	**V**	**W**	**Y**
**36**	A1	0.6	0.0	2.2	96.2	0.0	0.0	0.0	0.0	0.3	0.0	0.0	0.3	0.0	0.0	0.0	0.0	0.0	0.0	0.0	0.0
	B	0.0	0.0	0.8	98.5	0.0	0.1	0.0	0.0	0.2	0.0	0.0	0.0	0.0	0.1	0.0	0.0	0.0	0.0	0.0	0.0
	C	68.0	0.0	0.1	29.6	0.0	0.1	0.0	0.1	0.1	0.0	0.0	0.0	0.0	0.1	0.0	0.0	0.2	0.4	0.0	0.0
	D	0.6	0.0	1.4	97.2	0.0	0.0	0.0	0.0	0.3	0.0	0.0	0.0	0.0	0.3	0.0	0.0	0.0	0.0	0.0	0.0
**48**	A1	0.0	0.0	0.0	1.3	0.0	0.0	0.0	0.0	0.0	0.0	0.0	0.0	0.0	0.0	0.0	96.5	2.2	0.0	0.0	0.0
	B	0.0	0.0	0.0	0.1	0.0	0.0	0.0	0.0	0.0	0.0	0.0	0.0	0.0	0.0	0.0	97.5	2.2	0.0	0.0	0.0
	C	0.1	0.0	0.0	0.8	0.0	0.0	0.0	0.1	0.1	0.0	0.1	0.0	0.1	0.0	0.1	10.7	87.9	0.0	0.0	0.0
	D	0.0	0.0	0.0	0.3	0.0	0.0	0.0	0.0	0.0	0.0	0.0	0.0	0.0	0.0	0.0	97.5	2.3	0.0	0.0	0.0
**200**	A1	49.4	0.0	0.0	1.9	0.0	0.0	0.0	2.2	0.0	0.0	0.0	0.0	0.0	0.3	0.0	0.0	45.5	0.6	0.0	0.0
	B	20.7	0.0	0.0	2.9	0.0	0.0	0.0	9.9	0.5	0.1	0.1	0.1	0.0	0.0	0.4	0.5	62.8	0.7	0.0	0.0
	C	93.1	0.0	0.0	2.5	0.0	0.1	0.0	0.2	0.1	0.0	0.0	0.0	0.0	0.0	0.0	0.1	2.4	1.0	0.0	0.0
	D	7.4	0.0	0.0	0.8	0.0	0.0	0.0	48.7	5.7	1.4	2.0	0.0	0.0	0.3	0.8	0.6	29.5	1.4	0.0	0.0
**245**	A1	0.3	0.0	0.0	17.9	0.0	0.0	1.0	3.5	9.9	0.3	6.4	1.3	0.0	43.3	0.6	0.0	1.6	13.5	0.0	0.0
	B	0.4	0.0	0.0	10.7	0.0	0.1	0.1	3.0	6.8	0.9	12.0	0.3	0.0	2.5	0.2	0.0	2.9	59.2	0.0	0.0
	C	0.0	0.3	0.0	2.4	0.0	0.0	1.4	0.6	8.2	1.5	1.2	0.3	0.1	79.6	0.2	0.1	0.6	2.9	0.0	0.0
	D	0.0	0.0	0.0	9.9	0.0	0.0	0.3	3.1	47.6	0.6	9.3	1.4	0.0	11.0	0.3	0.0	13.9	0.8	0.0	0.0
**359**	A1	6.4	0.3	0.0	0.0	0.0	0.0	0.0	0.0	0.0	0.0	0.0	0.0	0.0	0.0	0.3	88.8	3.8	0.0	0.0	0.0
	B	0.1	0.1	0.1	0.1	0.0	92.3	0.0	0.0	0.0	0.0	0.0	0.0	0.0	0.0	0.1	6.9	0.1	0.0	0.0	0.0
	C	5.0	0.0	0.0	0.0	0.0	0.3	0.3	0.0	0.0	0.0	0.0	1.5	0.0	0.0	0.0	8.5	**83.7**	0.0	0.0	0.0
	D	0.0	0.0	0.0	0.0	0.0	91.2	0.0	0.0	0.0	0.0	0.0	0.0	0.0	0.0	0.0	8.5	0.0	0.0	0.0	0.0
**530**	A1	0.0	0.0	0.0	0.0	0.0	0.0	0.0	0.0	90.1	0.0	0.0	0.0	0.0	0.0	9.9	0.0	0.0	0.0	0.0	0.0
	B	0.0	0.0	0.0	0.0	0.0	0.0	0.0	0.0	92.6	0.0	0.0	0.0	0.0	0.1	6.7	0.0	0.0	0.0	0.0	0.0
	C	0.0	0.0	0.0	0.0	0.0	0.2	0.0	0.0	17.0	0.0	0.1	0.0	0.0	0.0	82.1	0.0	0.0	0.0	0.1	0.0
	D	0.0	0.0	0.0	0.0	0.0	0.0	0.0	0.0	96.3	0.0	0.0	0.0	0.0	0.0	3.1	0.0	0.0	0.0	0.0	0.0

Supplementary Table 1: The percentage prevalence of amino acids in various subtypes at the six locations shown in Figure 2A. The single letter amino acid code is used.

Near Full-Length Molecular Clones		
**Plasmid Backbone**	**RT Variants**	**EGFP Variants**
	**Amino Acid at Pos. 359**	**Frameshift Mutation (FSM) Position**
1.NL4-3-Δenv-EGFP<br>2. Indie-C1-Δenv-EGFP	Gly (WT for NL4-3)	None (WT)
	Thr (WT for Indie-C1)	4
	Ser	204

Plasmid Combinations		
**EGFP Variant Used for Transfection**		**GFP Fluorescence**
**Plasmid - 1**	**Plasmid - 2**	
WT	-	Yes
FSM 4	-	No
FSM 204	-	No
FSM 4	FSM 204	Yes

Supplementary Table 2: The composition of the viral variants shown in Figure 4A and the plasmid combinations that were used for virus production.
